# A review of Cushing's disease treatment by the Department of Neuroendocrinology of the Brazilian Society of Endocrinology and Metabolism

**DOI:** 10.20945/2359-3997000000014

**Published:** 2018-01-01

**Authors:** Márcio Carlos Machado, Maria Candida Barisson Vilares Fragoso, Ayrton Custódio Moreira, César Luiz Boguszewski, Leonardo Vieira, Luciana A. Naves, Lucio Vilar, Luiz Antônio de Araújo, Nina Rosa Castro Musolino, Paulo Augusto C. Miranda, Mauro A. Czepielewski, Monica R. Gadelha, Marcello Delano Bronstein, Antônio Ribeiro-Oliveira

**Affiliations:** 1 Universidade de São Paulo Universidade de São Paulo Hospital das Clínicas da Faculdade de Medicina Unidade de Neuroendocrinologia, Serviço de Endocrinologia e Metabologia São Paulo SP Brasil Unidade de Neuroendocrinologia, Serviço de Endocrinologia e Metabologia, Hospital das Clínicas da Faculdade de Medicina da Universidade de São Paulo (HCFMUSP), São Paulo, SP, Brasil; 2 Universidade de São Paulo Universidade de São Paulo Faculdade de Medicina de Ribeirão Preto Divisão de Endocrinologia e Metabologia Ribeirão Preto SP Brasil Divisão de Endocrinologia e Metabologia, Faculdade de Medicina de Ribeirão Preto, Universidade de São Paulo (FMRP-USP), Ribeirão Preto, SP, Brasil; 3 Universidade Federal do Paraná Universidade Federal do Paraná Hospital de Clínicas Serviço de Endocrinologia e Metabologia (SEMPR) Curitiba PR Brasil Serviço de Endocrinologia e Metabologia (SEMPR), Hospital de Clínicas, Universidade Federal do Paraná (UFPR), Curitiba, PR, Brasil; 4 Universidade Federal do Rio de Janeiro Universidade Federal do Rio de Janeiro Hospital Universitário Clementino Fraga Filho Serviço de Endocrinologia Rio de Janeiro RJ Brasil Serviço de Endocrinologia, Hospital Universitário Clementino Fraga Filho, Universidade Federal do Rio de Janeiro (HUCFF-UFRJ), Rio de Janeiro, RJ, Brasil; 5 Universidade de Brasília Universidade de Brasília Hospital Universitário de Brasília Serviço de Endocrinologia Brasília DF Brasil Serviço de Endocrinologia, Hospital Universitário de Brasília, Universidade de Brasília (UnB), Brasília, DF, Brasil; 6 Universidade Federal de Pernambuco Universidade Federal de Pernambuco Hospital de Clínicas Serviço de Endocrinologia Recife PE Brasil Serviço de Endocrinologia, Hospital de Clínicas, Universidade Federal de Pernambuco (UFPE), Recife, PE, Brasil; 7 Endoville Joinville SC Brasil Endoville, Joinville, SC, Brasil; 8 Universidade de São Paulo Universidade de São Paulo Hospital das Clínicas da Faculdade de Medicina Divisão de Neurocirurgia Funcional São Paulo SP Brasil Divisão de Neurocirurgia Funcional, Hospital das Clínicas da Faculdade de Medicina da Universidade de São Paulo (HCFMUSP), São Paulo, SP, Brasil; 9 Santa Casa de Belo Horizonte Serviço de Endocrinologia Belo Horizonte MG Brasil Serviço de Endocrinologia, Santa Casa de Belo Horizonte, Belo Horizonte, MG, Brasil; 10 Universidade Federal do Rio Grande do Sul Universidade Federal do Rio Grande do Sul Hospital de Clínicas de Porto Alegre, Faculdade de Medicina Serviço de Endocrinologia Porto Alegre RS Brasil Serviço de Endocrinologia, Hospital de Clínicas de Porto Alegre, Faculdade de Medicina da Universidade Federal do Rio Grande do Sul (UFRGS), Porto Alegre, RS, Brasil; 11 Universidade Federal de Minas Gerais Universidade Federal de Minas Gerais Hospital de Clínicas Serviço de Endocrinologia Belo Horizonte MG Brasil Serviço de Endocrinologia, Hospital de Clínicas, Universidade Federal de Minas Gerais (UFMG), Belo Horizonte, MG, Brasil

**Keywords:** Cushing's disease, Cushing's syndrome, treatment

## Abstract

The treatment objectives for a patient with Cushing's disease (CD) are remission of hypercortisolism, adequate management of co-morbidities, restoration of the hypothalamic-pituitary-adrenal axis, preservation of fertility and pituitary function, and improvement of visual defects in cases of macroadenomas with suprasellar extension. Transsphenoidal pituitary surgery is the main treatment option for the majority of cases, even in macroadenomas with low probability of remission. In cases of surgical failure, another subsequent pituitary surgery might be indicated in cases with persistent tumor imaging at post surgical magnetic resonance imaging (MRI) and/or pathology analysis of adrenocorticotropic hormone-positive (ACTH+) positive pituitary adenoma in the first procedure. Medical treatment, radiotherapy and adrenalectomy are the other options when transsphenoidal pituitary surgery fails. There are several options of medical treatment, although cabergoline and ketoconazole are the most commonly used alone or in combination. Novel treatments are also addressed in this review. Different therapeutic approaches are frequently needed on an individual basis, both before and, particularly, after surgery, and they should be individualized. The objective of the present review is to provide the necessary information to achieve a more effective treatment for CD. It is recommended that patients with CD be followed at tertiary care centers with experience in treating this condition.

## INTRODUCTION

Cushing's syndrome is associated with a high mortality risk (1-10). A meta-analysis has found a standardized mortality ratio (SMR) of 2.22 (range, 1.45 – 3.41; confidence interval, CI, 95%) in patients with Cushing's syndrome compared to the general population (7). The major causes of mortality in these patients are cardiovascular diseases (ischemic heart disease and cerebrovascular diseases), diabetes mellitus (secondary to hypercortisolism), and infections (due to immunosuppression).

Clearly, the mortality rate is influenced by the disease activity. The SMR is higher in patients with persistent disease when compared to patients in clinical remission of the hypercortisolism: 5.50 (range, 2.69 – 11.26) vs. 1.20 (range, 0.45 – 3.18), respectively (7).

Nevertheless, even after the resolution of hypercortisolism, there may not be a complete reversal of cardiovascular risk factors or, alternatively, a complete reversal may take more than 5 to 6 years to occur (8,11,12). In addition, patients with active Cushing's disease (CD) present with poorer quality of life and lower scores might persist even after surgical remission (13).

One factor contributing to the onset and progress of associated co-morbidities is the time spent since the recognition of the disease, its diagnostic confirmation (involving complex and expensive laboratoty and imaging studies), and correct definition of the etiology (1,14-17). Accordingly, a recent study showed increased mortality in patients with longer exposure to hypercortisolism (8).

Thus, to improve the prognosis of patients with Cushing's syndrome and to help reverse morbidities, it is important to identify the disease and to achieve eucortisolism as soon as possible.

The objective of the present review is to provide the necessary information to achieve a more effective treatment of CD. The specific therapeutic approach of the several co-morbidities associated with CD is beyond the scope of this review. The present manuscript highlights the importance of centers of excellence with a highly experienced multidisciplinary team for longterm follow-up of these patients

## TREATMENT

The goals of the treatment of CD are as follows: (i) remission of hypercortisolism, (ii) adequate management of co-morbidities and cardiovascular risk factors, (iii) restoration of the hypothalamic-pituitary-adrenal axis, (iv) preservation of fertility and maintenance of pituitary function and (v) improvement of visual defects in cases of macroadenomas with suprasellar extension. However, it is common that one or more objectives need to be sacrificed to achieve remission in a patient (18).

Although surgical treatment generally results in high remission rates at short term in series from specialized centers (~70-80%), recurrence is observed in a substantial proportion of patients who then need other therapies to control hypercortisolism.

### Surgical treatment

Despite advances in drug treatment and progress in other therapeutic modalities, such as stereotactic radiotherapy, surgical treatment is still the principal definitive treatment for CD (19).

Some recommendations regarding the preoperative management of patients with CD should be taken into account. Due to a higher risk of cardiovascular complications, such as coronary heart disease, it is important to conduct a careful cardiac assessment of these patients. However, the risk stratification does not differ from that in individuals without Cushing's syndrome. There is also increased risk of thromboembolic events, both pre- and postoperatively (PO) (20-25). Thus, the analysis of coagulation factors is very important, although there are no formal guidelines to define which factors should be analyzed in addition to those traditionally considered (i.e., thrombin time, prothrombin time, thromboplastin activated time, platelets) or which coagulation protocol should be recommended before and after surgery (26). In patients treated with antiplatelet drugs (to prevent ischemic events), antiplatelet therapy should be withdrawn at least seven days before surgery.

Usually, drug treatment for hypercortisolism is unnecessary during the preoperative period. Nevertheless, in some cases, it is necessary to initiate a specific medical treatment during the preoperative period, for instance, in patients with more severe disease and increased preoperative risk and when surgical treatment can not be immediately performed. The most commonly used approach is therapy with adrenal steroidogenesis inhibitors, particularly ketoconazole, which has a shorter half-life than the dopaminergic agonist cabergoline, which acts on corticotroph tumors and causes less interference with cortisol measurements during the PO period (27).

Many factors influence transsphenoidal surgery remission rates, including characteristics of the pituitary adenoma (e.g., tumor size, location, extension, aggressiveness, histological type and radiological identification), surgical procedure (e.g., the quality of the equipment and surgical technique), variability of the remission criteria used, and, particularly, the surgeon (e.g., experience, surgical identification of the tumor) (28).

All factors mentioned above contribute to the wide range of PO remission rates reported on by numerous case studies. In general, the mean remission rate ranges from 70% to 90% in several reviews (19,27,29,30). However, within the same case series, the remission rate may vary, depending on the subset of the patient analyzed, e.g., whether patients had a micro or a macroadenoma, whether tumor was identified on preoperative pituitary magnetic resonance imaging (MRI) or during surgery; and whether patients had been previously submitted to bilateral and simultaneous petrosal sinus sampling (BIPSS). Importantly, recurrence may occur several years later.

In cases of macroadenoma (≥ 10 mm), the remission rate ranges from 50% to 70% (27) and is generally lower than the microadenoma remission rate (19). These percentages can vary depending on the size and, particularly, the degree of invasion of the adenoma into the adjacent tissues. A study performed on a small sample (n = 40) identified tumor size as the main factor responsible for post surgical remission rate. The authors observed remission rates of 84% for microadenomas (21/25), 92% for macroadenomas (11/12; mean of 15 mm; only 2 with invasion) and no remission for patients without visible tumor during surgery (n = 3) (31).

The remission rate in patients submitted to BIPSS is also lower than that observed in visible microadenomas on MRI and ranges from 50% to 70% (27,32). Interestingly, a study that specifically assessed remission in these cases did not show any difference between positive and negative MRI findings (33). In this subset, the main factor influencing remission rate was the intraoperative identification of the tumor.

Other predictive factors of a better surgical outcome include absence of invasion of the duramater or cavernous sinuses, histological confirmation of adrenocorticotropic hormone-positive (ACTH+) adenoma, low serum cortisol levels during the PO period, and prolonged secondary adrenal insufficiency (> 12 months) (27).

In pediatric patients, the number of reported cases is lower, but remission rates are similar or slightly increased in some cases, ranging from 83% to 98% (34,35). A recent study identified histopathology with an ACTH+ pituitary adenoma and no invasion as early predictors of remission in children. Young age, smaller tumors and no invasion of duramater or cavernous sinuses were predictive of long-term remission (35).

Almost all patients undergo pituitary surgery via transsphenoidal endonasal approach, including those with macroadenoma (29,30). Craniotomies are the exception and are indicated in rare selected cases. The most studied is the microscopic technique, but the endoscopic approach has been increasingly used in the last decade. The remission rate is similar for both techniques, particularly in the case of microadenomas (30,36-40). For macroadenomas and invasive tumors, the endoscopic technique has a potential advantage in offering a greater angular field of view, and therefore visualizing and removing tumors impinging the cavernous sinus or extending beyond the sellar boundaries. Other techniques that can improve intraoperative tumoral localization, such as ultrasonography, neuronavigation, rapid pathological and/or hormonal analysis and intraoperative MRI, are not available in most centers and therefore their usefulness cannot be properly evaluated so far.

Adenomectomy is the most performed surgery, although exploration of the entire gland is justified in most cases. In patients with no identified tumor, an ipsilateral hemi-hypophysectomy (partial hypophysectomy) is usually performed in the side suggested by BIPSS with lower remission rates. Total hypophysectomy is rarely justified due to a limited remission rate in such cases and expected hypopituitarism (27).

Pituitary surgery has a low mortality rate (0% to ≤ 1.5%), (29,41) comparable to the outcomes observed in simpler surgical procedures. The most common complications are endocrine: transient diabetes insipidus in 3% to 9% of patients (polyuria and/or hypernatremia), hyponatremia that can result from secondary adrenal insufficiency, particularly in patients not taking glucocorticoids, or syndrome of inappropriate antidiuretic hormone secretion (SIADH), and other pituitary deficiencies (growth hormone deficiency, hypogonadotropic hypogonadism, central hypothyroidism). Other complications include cerebrospinal fluid fistula (< 8%), bleeding or hematomas (range, 1-6%), epistaxis, infections (particularly sinusitis), and thromboembolic events (29). Due to the risk of thromboembolic events, it is recommended to perform active prophylaxis, including pneumatic compression of the lower limbs, low-molecular-weight heparin treatment as soon as possible (24h after surgery), and early mobilization during the hospital stay. However, there is no current specific anticoagulation protocol for Cushing's disease. The rates of complications derived from microsurgery and endoscopic techniques are similar.

### Criteria for remission, glucocorticoid replacement therapy, and prediction of recurrence risk

Several clinical and laboratory criteria are used to define PO remission, but there is no consensus or ideal method that guarantees a recurrence-free followup period (29). However, a patient who develops adrenal insufficiency with very low serum cortisol levels (< 2 mg/dL) and requires glucocorticoid replacement therapy clearly exhibits PO remission. However, these “rigid” laboratory criteria are not found in up to 20% of patients who show long-term remission and exhibit “normal” PO cortisol levels (42). Other factors used to define post surgical remissions are reversal of hypercortisolism, need of glucocorticoid replacement therapy and normalization of cortisol parameters, particularly urinary free cortisol (UFC) lasting at least 6 months after surgery.

Adrenal insufficiency is not clear in all cases, particularly when early glucocorticoid replacement therapy is routinely performed or in previously treated patients who undergo surgery and who exhibit eucortisolism. The symptoms that indicate adrenal insufficiency are asthenia, appetite loss, nausea, skin peeling, joint and muscle pain, weight loss, low blood pressure and/or postural hypotension. Mild hyperthermia, a transient increase in TSH levels, and hyponatremia might also occur. Although ACTH also stimulates aldosterone secretion, it should be highlighted that severe hypotension and hyperkalemia are not common due to the integrity of the renin-angiotensin-aldosterone system.

The most studied and utilized laboratory parameter is serum cortisol. In a recent guideline, a cortisol level < 5 µg/dL in the first PO week was stated as indicative of remission (19). Other authors have attempted to identify a more accurate “ideal” cortisol level (43). However, it is known that even undetectable levels of serum cortisol are not a guarantee of long-term remission (44), and an important study observed recurrence in 20% of patients at 5 years, even among those with cortisol levels < 2 µg/dL (45). Thus, more important than determining an “ideal value” is to understand that there are different levels of recurrence associated with serum cortisol values, as follows: < 2, low risk; 2 – 5, intermediate risk; > 5 µg/dL, high risk (46). Another important finding is that 5.6% of patients present a gradual decline in cortisol levels after the first week (“late remission”) (47). For this reason, other cortisol samples must be collected, particularly during the first PO month. The two most cited explanations for this fact are the persistence of cortisol secretion due to chronically stimulated adrenal glands and the subsequent post surgical necrosis of corticotropic tumor cells. Interestingly, one study has shown increased long-term recurrence in patients from this late remission subgroup (47).

A very important factor for the analysis of serum cortisol levels in the PO period is glucocorticoid replacement therapy. Generally, two replacement strategies have been used and they do not include glucocorticoids during anesthetic induction. In one strategy, routine replacement therapy is not performed during the immediate PO period and in the initial days. Despite the short half-life of cortisol (range, 50–70 minutes) and intense reduction of its serum concentration after a successful adenoma removal, the patient does not usually show adrenal insufficiency too early – 24-48 hours – after surgery (48-50). Thus, the measurement of morning cortisol or a 6/6-hour curve starting in the immediate PO period is performed during the first days, and glucocorticoid replacement is initiated only after suggestive symptoms of adrenal insufficiency (with measurement being performed immediately before) and/or when low levels of cortisol are detected (< 5 µg/dL). Endocrinologists should closely assess the patient, if possible, until replacement is initiated. In this strategy, one advantage is that serum cortisol measurements are not influenced by exogenous corticosteroids. The second strategy consists of initiating routine glucocorticoid replacement therapy during the immediate PO period, preferably with short half-life corticoids, such as hydrocortisone (immediate PO, 25–50 mg intravenously three times per day), followed by oral hydrocortisone (from the 1^st^ PO day forward: 20 mg early in the morning, 10 mg at 2 PM). In this way, serum cortisol measurements are performed only in the morning from 8 to 9 AM on any given day, under fasting condition, 18 to 24 hours after the last dose. The main advantages of this strategy are (i) easy applicability; (ii) patient safety; (iii) a lower incidence of adrenal insufficiency symptoms, although relative adrenal insufficiency can still occur with replacement therapy; (iv) reduced suppression of the hypothalamic-pituitary-adrenal axis (good for the pediatric population) compared with longer half-life drugs, such as prednisone or dexamethasone; and (v) easy measurement of serum cortisol, which allows the physician to observe the progressive increase of serum cortisol levels along with the recovery of the axis, which occurs 6 to 18 months PO.

However, although it is the most used glucocorticoid in this clinical setting, hydrocortisone is commercialized in Brazil in just one tertiary center (hydrocortisone, 20 and 5 mg tablets). The advantage of using dexamethasone (dose: 0.25 – 1 mg once daily; tablets with 0.5, 0.75 and 4 mg) is that it does not usually interfere with the serum and urine cortisol measurement, and it has been used in some centers (29). However, due to its longer half-life, even when used at low doses, it is not possible to exclude the suppression of the axis, potentially leading to underestimated cortisol levels. The only advantage of prednisone (dose: 2.5 – 5 mg once daily; tablets, 5 and 20 mg) is the diffuse availability of the product; however, this drug can cause interference with the cortisol measurement and may suppress the axis with chronic use, although the risk of suppressing the axis is lower when compared to dexamethasone. Prednisone should be withdrawn at least 48 h before serum and/urine cortisol measurements, leading to an increased need of observation for the risk of adrenal insufficiency. Recently, another form of oral dualrelease hydrocortisone has been studied in patients with adrenal insufficiency (51). However, there is no data on advantages of these formulations in this subgroup of PO patients with CD in order to normalize the hypothalamic pituitary adrenal axis.

In addition to serum cortisol, other laboratory criteria have been used, albeit infrequently, to define short-term remission (40). Among these criteria are lower than normal UFC (47), lower than normal plasma ACTH (52) and cortisol suppression after a low dose of dexamethasone (15).

Among these parameters, ACTH measurement is currently the most studied and is primarily used to predict the risk of long-term recurrence (49,53). A study that analyzed patients in initial PO remission (serum cortisol < 3 µg/dL) showed lower PO ACTH in patients with long-term remission compared to those with recurrent disease (11.9 vs. 34.3 pg/mL, *p* < 0.0001, respectively) (49).

One study investigated late-night salivary cortisol and found this parameter to be more accurate in predicting PO remission in comparison to serum cortisol and UFC (54). Late-night salivary cortisol was measured starting from 6 months PO. Further studies are necessary to show the utility of this method in assessing initial remission. However, there are studies showing a good utility of this method for earlier diagnosis of recurrence, even before the UFC (55,56).

Other methods are used to predict the risk of long-term recurrence. Primary among these methods are measurements of ACTH or cortisol following administration of corticotropin-releasing hormone (CRH) (46), metyrapone, thyrotropin-releasing hormone (TRH), luteinizing hormone-releasing hormone (LHRH), loperamide and desmopressin. The rational of these tests derives from the incapacity of normal corticotropes, suppressed by hypercortisolism, to secrete ACTH. In this way, early PO responses suggest the presence of residual tumor cells and, consequently, an increased risk of recurrence. The response to desmopressin is the most used test, with several studies showing similar results (57-64). One of the major problems with this test is the definition of ACTH and cortisol responses after desmopressin administration in the PO period. Several authors have used criteria similar to those used in the preoperative period (i.e., serum cortisol increased by > 20% and ACTH increased by > 30% to 50%) (57-61,63,64). These definitions, however, may overestimate any observed increase; for example, a change in serum cortisol from 1 to 2 µg/dL could correspond to an increase of 100%. A previous study assessed the risk of recurrence if the variation of serum cortisol is > 7 µg/dL (variation (Δ): peak minus time 0) after IV desmopressin 10 µg administration approximately 2 weeks PO. This method had a specificity of 100% and a sensitivity of 33%. It should be noted that in this previous study, only patients with low serum cortisol (< 6 µg/dL approximately 6 days PO) were subjected to the test, excluding patients with a risk of recurrence due to increased PO cortisol levels (62). Another factor to be kept in mind as associated to lower risk of recurrence is the higher length of postoperative glucocorticoid replacement, such as more than 12 months (41).

### Surgery following initial surgical failure

After an initial surgical failure, the clinical case must be entirely reviewed. A diagnosis of CD should be confirmed by histopathological examination of the pituitary adenoma, which should be ACTH-positive on immunohistochemistry. If no adenoma has been found and the pituitary gland is reported as normal, then the diagnosis might be confirmed through PO remission or through the central to peripheral ACTH gradient at BIPSS. BIPSS might be performed with the sole objective of confirming a central origin not yet proven, considering that lateralization cannot be trusted to predict tumor localization. Another important issue is the description of the surgery as reported or registered by the surgeon. For example, a report of partial tumor resection due to invasion of the cavernous sinus in a macroadenoma patient limits the indication for subsequent surgery (29). The confirmation of pituitary adenoma is also important in deciding upon a new pituitary surgical procedure. This should be evaluated with a new pituitary MRI performed at least 3 months after transsphenoidal surgery showing a residual tumor (65). These aspects need to be carefully analyzed before referring patients for another pituitary surgical procedure, especially if the first operation was not performed by an experienced surgeon (29). The remission rate is lower than observed for the first surgery, ranging from 40% to 70% (27,66,67), a rate that is similar to that obtained with current medical treatment. In addition, it is important to highlight that the rate of complications, including cerebrospinal fluid fistula and hypopituitarism, is higher when compared to the first surgery.

Some authors recommend early re-operation at 3 to 15 days after initial surgical failure, defined by serum cortisol > 2 µg/dL (53,68-70). However, as late remission can occur in 5.6% of patients 30 to 50 days after the initial surgery (47), this strategy is not commonly used.

### Medical treatment

Medical treatment can be classified as primary or secondary. Primary therapy is used to lower cortisolemia and improve preoperative clinical conditions, or in cases of surgical contraindication or refusal, or before other definitive approaches. Secondary treatment after surgical failure is much more common, and is indicated for patients with relapse and no indication for a new surgery, as well as in patients who undergo pituitary radiotherapy. The drugs are classically divided into three classes: a) acting on the ACTH-secreting tumor, b) adrenal steroidogenesis inhibitors, and c) cortisol receptor antagonists. The first two classes comprise several drugs, some of which are currently in use and several others are not available or are under development. The limited number and availability of these drugs reflect the difficulty of controlling cortisol levels in CD patients. No ideal treatment is available, as reviewed in several recent articles (18,22,23,71-80).

### Drugs acting on the corticotropic tumor

#### Cabergoline

Due to the very frequent expression of dopaminergic subtype 2 receptors (DRD2) on the surface of tumor cells in several types of pituitary adenomas, dopamine agonists, particularly cabergoline, have been used to treat prolactinomas (first option), acromegaly (adjuvant), clinically non-functioning adenomas and CD (adjuvant).

A study on corticotropic tumors elegantly demonstrated the expression of DRD2 in more than 80% of tumor samples, which exhibit binding affinity and *in vitro* inhibition of ACTH secretion in response to dopamine agonists (50).

For the treatment of CD, bromocriptine was initially used with limited efficacy and common side effects due to the need of high doses (range, 3.75 – 30 mg). Thus, the use of bromocriptine is no longer justified. Cabergoline, a better-tolerated and more potent and specific DRD2 receptor agonist, has been assessed for efficacy in some studies (50,81-85). UFC normalization, the primary endpoint for most studies, was observed in 25 – 40% of the 72 patients analyzed (4 studies with at least 10 cases), with mean dose of 3 mg/week (range, 1 – 7) and an average treatment period of 18 months (range, 3 – 60) (50,81-83). No long-term response predictor has been identified and there have been few cases studying the correlation between the *in vivo* response and DRD2 receptor tumor expression (50). The efficacy of cabergoline declines with increasing treatment duration, an effect that is primarily due to tachyphylaxis, which occurs due to unknown mechanisms and is observed in 18 – 30% of cases (81,82), even with prolonged use (1 – 5 years) (81,82). Cabergoline therapy is usually initiated at 1 mg/week (0.5 mg twice per week, at night; tablets, 0.5 mg), with a monthly increase of 1 mg if UFC normalization does not occur. One study defined unresponsiveness as a reduction in UFC lower than 25% after 3 months of treatment with increasing doses (81). A reduction in tumor size/volume was poorly assessed in previous studies. A reduction of at least 25% of the tumor size occurred in 50% of cases in one study (81), although case reports have demonstrated significant reductions (86,87). There is a recommendation for monitoring cardiac valves by echocardiogram during cronic use of cabergoline due to potential risk of thickening described with larger doses of cabergoline in Parkinson's patients (88). However, there are no data on echocardiographic changes after prolonged use in Cushing's patients. Finally, although studies have shown the effectiveness of cabergoline in a subset of patients with CD, its use is off-label, and it is not approved for CD treatment in Brazil.

#### Pasireotide

The first-generation somatostatin analogues octreotide and lanreotide, traditionally used to treat patients with acromegaly, are not indicated for the treatment of CD due to the low expression of the subtype 2 somatostatin receptor (SSTR2) as a consequence of down regulation by hipercortisolism in corticotrope tumors (89,90). The development of new somatostatin analogues with a different affinity profile to SSTR subtypes has opened new therapeutic possibilities. Pasireotide is the first specific drug approved for the treatment of CD in Europe, USA and recently in Brazil (ampules for subcutaneous use: 300, 600 and 900 mg). In comparison to first-generation somatostatin analogues, pasireotide has increased affinity to SSTR1 (> 30 times), SSTR3 (> 3 times) and, particularly, SSTR5 (> 40 times) (27), which is the most expressed receptor in tumor corticotropes (91). This new analogue has been tested both *in vitro* (92,93) and *in vivo* studies (94). The core study employed in the pasireotide approval was published in 2012 (95). This was a prospective, multicenter, double-blind study on the use of pasireotide in patients with persistent or recurrent CD or naïve patients who could not undergo surgical treatment. Two doses were tested (600 or 900 µg SC twice per day) for 12 months, and a total of 162 patients were assessed. A significant UFC reduction (at least > 50%, primary endpoint) was observed in 49% of patients at 6 months, with UFC normalization in 28.8% of patients who were treated with 900 µg of pasireotide, a response that was maintained up to 12 months with no relapses during this period. A subsequent extension study showed a similar and sustained response after 24 months (96) and even in longer studies up to 72 months (73,91,97).

The therapeutic response to pasireotide depends on the intensity of hypercortisolism. Complete response was noted in 50% of patients with baseline UFC up to twice the upper limit versus 8% in those with baseline UFC > 5 times the upper limit (95). In addition, an early response at 2 to 3 months predicted a sustained response at 12 months. Parameters such as weight, blood pressure, lipid profile and quality of life improved in patients with reduced UFC, even when normalization was not achieved. An analysis of tumor volume was performed in a subset of patients and showed a reduction of 43.8% (compared to initial volume) at 12 months in patients who are treated with 900 mg. Side effects were very common and similar to those of other somatostatin analogues (i.e., diarrhea, nausea, cholelithiasis, mild increase of liver enzymes, bradycardia, and others). At 12 months, 73% of patients experienced adverse events related to hyperglycemia, but most of the events were considered mild to moderate (95). These events are due to a concomitant inhibition of insulin, glucagon-like peptide 1 (GLP1) and glucose-dependent insulinotropic polypeptide (GIP), as well as to only a slight inhibition of glucagon (43,98). Thus, in addition to metformin (usually used in CD patients), dipeptidyl peptidase 4 (DPP4) inhibitors or GLP1 analogues should be the first options to treat hyperglycemia in this situation. Another undesired effect observed in a short-term study (80 days) was a reduction in insulin-like growth factor 1 (IGF1) levels to lower than normal in > 50% of patients (99).

### Adrenal steroidogenesis inhibitors

#### Ketoconazole

Ketoconazole is one of the most prescribed drugs for the treatment of CD, despite being used off label. This compound is an imidazole antifungal drug that reversibly inhibits adrenal steroidogenesis through the action of several enzymes (i.e., cholesterol desmolase, 17αOH and 11β-hydroxylase). Ketoconazole also inhibits androgen production resulting in hypogonadism in men (gynecomastia, decreased libido and erectile dysfunction); in women, in contrast, it might improve hyperandrogenism.

Two studies published in 2008 and 2014 provided the best assessment of ketoconazole use in CD (100,101). In the oldest study, 38 cases were analyzed and 6 series were reviewed, totalizing 99 patients. The series included patients who had undergone previous radiotherapy (102), small numbers of cases (6 – 8 cases) (103-107), or short-term follow-ups (range, 15 – 30 days) (104-107). Of the 38 patients, 51.5% achieved a normal UFC with a mean treatment period of 22 months (6 – 72) and a mean dose of 529 mg/day (range, 200 – 1000 mg). Five patients (13%) interrupted treatment due to side effects (nausea, diarrhea, and increased levels of transaminases by five-fold in one patient) during the first week. A few cases without visible tumors were primarily treated with ketoconazole. Onethird of these patients (5/15) showed visible lesions during follow-up (after 12 – 30 months) and were then submitted to surgery (101). No tumor progression occurred in patients who already had a lesion visible by MRI, although the study did not report the proportion of micro- and macroadenomas. Similar results were found in the more recent multicenter retrospective study with a larger group of 200 patients. Normal UFC was observed in 49.3% of patients with mean dose of 600 mg/day during mean time of observation of 24 months (100).

Another study showed similar control of disease activity in 9/17 patients (53%) during the preoperative period of patients with Cushing's syndrome (pituitary or adrenal in 85% of cases) treated with 200 – 1000 mg/day over a mean period of 4 months (108). Usually, treatment begins with 400 mg/day (200 mg twice daily; tablets: 200 mg), with the medication not taken near meals, as an acidic pH is needed for absorption. Accordingly, the use of proton pump inhibitors decreases drug availability. The dose is increased monthly, up to 1200 mg/day, to achieve UFC normalization, although it is uncommon to reach the maximum dose. Similar to cabergoline, tachyphylaxis may occur in up to 33% of patients following prolonged use of ketoconazole (101,108). Mild side effects are relatively common and include headache, nausea, and rash. Another important side effect is increased levels of hepatic transaminases by as much as 3 times the upper limit. Such increase is usually asymptomatic and reversible with drug interruption or dose reduction. Thus, it is important to monitor hepatic transaminases during the first month of treatment and thereafter. Idiosyncratic severe hepatic insufficiency has been described on rare occasions (19,109).

#### Metyrapone

Metyrapone is used to assess the sufficiency of the hypothalamic-pituitary-adrenal axis and to treat CD. A reduction of hypercortisolism is achieved by blocking adrenal steroidogenesis via the inhibition of 11β-hydroxylase. This enzyme converts 11-desoxicortisol (compound S) to cortisol, and treatment with metyrapone can result in a rebound of ACTH levels (73). Treatment usually begins at 250 – 500 mg, 3 – 4 times per day, with a maximum dose of 4 – 6 g/day (capsules, 250 mg). In addition, the drug acts rapidly (from hours to days). Due to ACTH rebound and a shift in the production of other steroids, metyrapone increases androgen production and commonly causes hirsutism and acne. This drug may also cause mineralocorticoid effects, such as hypertension and hypokalemia. In general, shortterm studies show control of the cortisol excess in a significant number of patients (73). A study showed control in 75% of patients treated with a mean dose of 2250 mg/day (110). One study assessed the use of metyrapone during the preoperative period in patients with Cushing's syndrome (85% pituitary) and showed UFC control in 26% of patients (6/23) treated with 750 – 4500 mg/day for an average of 4 months (108). In the larger multicenter retrospective study, normal UFC was found in 43% of Cushing's syndrome patients from all etiologies (CD, Ectopic ACTH syndrome (EAS), adrenal diseases) in a mean of 8 months (3 days – 12 years) (111). Long-term studies with a large number of CD cases are necessary to better assess the effects of this drug. In addition, this drug is not available in Brazil. Metyrapone is currently available in USA and Europe.

#### Etomidate

Etomidate is an intravenous anesthetic (imidazole carboxylate derivative) that decreases cortisol levels by inhibiting 11β-hydroxylase (112,113). The main advantage of etomidate is its rapid time of action, allowing reduction or normalization of serum cortisolin less than 24 hours. Thus, etomidate is useful for severe cases of Cushing's syndrome, generally patients with ectopic ACTH syndrome (EAS). Treatment is performed in hospitalized patients, especially in intensive care units, due to clinical severity and the need for close monitoring, although the dose used is usually safe and does not cause severe sedative effects. Treatment assessment is primarily performed by the measurement of serum cortisol (113), and care should be taken to avoid adrenal insufficiency. ‘Block and replace’ therapy with hydrocortisone IV can be used. Treatment is performed by continuous intravenous infusion and may consist of an initial bolus of 0.03 mg/kg followed by 0.1 – 0.3 mg/kg/hour (ampules, 2 mg/mL). Intermittent use for several hours with periodic intervals has been described. A review published in 2012 including 18 studies (mostly case reports) with a total of 12 patients with CD found cortisol normalization in virtually all cases when used from 5 hours to 56 days (113).

#### Mitotane

Also known as o,p′-DDD (dichloro-diphenyl-dichloro-ethane), mitotane is an oral chemotherapy used to treat patients with adrenal carcinoma. Mitotane is considered an adrenolytic compound due to mitochondrial toxicity that causes cellular necrosis. In addition, mitotane inhibits the adrenal production of cortisol by acting on enzymes involved in steroidogenesis (i.e., 11β-hydroxylase and cholesterol desmolase) (114). Mitotane is a lipophilic drug, has a slower mechanism of action than other inhibitors, and has a very long half-life due to storage in adipose tissue (range, 18 – 159 days). The established dose for the treatment of adrenal cancer is high (approximately 8 – 12 g/day), and the effective dose is verified by mitotane levels > 14 – 20 µg/mL. However, for the treatment of CD, lower, non-adrenolytic doses are prescribed, i.e., approximately 2 – 4 g/day (115), depending on the patient's profile and UFC. Usually, treatment is initiated at 500 mg at bedtime, with doses increasing every 1 – 4 weeks (according to tolerance to treatment) up to 2 – 3 g/day in fractionated doses at meals (tablets, 500 mg). In contrast to the effective dose for adrenal cancer treatment, there is no target mitotane concentration for CD, and UFC monitoring is the most important parameter. In one study, mitotane levels > 8.5 µg/mL were associated with normal UFC levels during follow-up (114). Use of mitotane is limited due to relatively common and severe side effects, such as nausea, vomiting, anorexia, rash, diarrhea, ataxia, gynecomastia, arthralgia, leukopenia, hepatotoxicity and hypercholesterolemia. In addition, mitotane may cause adrenal insufficiency, which can occasionally be underestimated due to an increase in cortisol binding globulin (CBG) levels. Due to increased corticoid metabolism, higher doses might be needed for adrenal insufficiency replacement. One study that assessed the use of mitotane in 76 patients with CD showed UFC normalization in 72% of patients with a mean treatment duration of 6.7 months (range, 5.2 – 8.2; mean dose, 2.6 ± 1.1 g/day) (114). Similar to the ketoconazole study (101), 25% of cases without a visible pituitary tumor developed a visible lesion during follow-up, which allowed patients to undergo surgical treatment (114). Due to limited availability, difficult management and high cost, this option is rarely used in Brazil for the treatment of CD.

### Cortisol receptor antagonist mifepristone

Mifepristone is an antiprogestogen that, at high doses, rapidly and competitively antagonizes the cortisol receptor, resulting in a rebound increase of ACTH and cortisol plasma levels (116). Thus, monitoring of mifepristone therapy in Cushing's syndrome should be performed using clinical and biochemical parameters such as serum glucose, and not ACTH or cortisol levels. Mifepristone was approved in 2012 in the USA to control hyperglycemia (diabetes mellitus or glucose intolerance) in patients with endogenous Cushing's syndrome (tablets, 300 mg). The approval was based mainly on the results of the SEISMIC trial (71), a prospective, multicenter, 24-week study of patients with endogenous Cushing's syndrome, and diabetes mellitus/glucose intolerance or isolated high blood pressures, who were unresponsive to other therapies (71). The trial involved 50 patients: 43 with CD, 4 with EAS, and 4 with adrenal carcinoma. All of the patients received initially 300 mg/day, and the dose was increased to 600, 900, and 1200 mg/day every 4 weeks if clinical improvement was not observed. The primary endpoints were (i) a decrease in the area under the curve (AUC) of glucose of at least 25% on the 75 g oral tolerance test and (ii) a reduction by > 5 mmHg of diastolic blood pressure. With a mean dose of 600 mg/day, improvement in glycemia (AUC) was observed in 60% of patients (mean reduction, 36%), and the HbA1c levels decreased from 7.43 ± 1.52 to 6.29 ± 0.99%. Diastolic blood pressure improved in 38.1% of patients. In addition, improvements were observed in weight (-5.7%), waist circumference, and insulin sensitivity. The main side effects, primarily mild or moderate, were nausea, fatigue, headache, hypokalemia (effect of cortisol on mineralocorticoid receptor), arthralgias and endometrial thickening/menorrhagia. Others concerns about the use of mifepristone is the adrenal insufficiency not biochemically detected but amenable to be treated with high dose of dexamethasone while withholding mifepristone, and the possible risk of tumor enlargement recently published (117).

### Combination therapy and perspectives

Given that the control rate is limited with the currently used drugs, especially in patients with severe Cushing's syndrome, combinations of different medications have been used as an alternative approach to control hypercortisolism (118). Combination therapy can be performed with medications from the same therapeutic class (e.g., combined use of steroidogenesis inhibitors) or from different classes (e.g., cabergoline + ketoconazole).

### Combined use of adrenal steroidogenesis inhibitors

At least two recent studies assessed the effect of the combined use of adrenal steroidogenesis inhibitors. Using ketoconazole and metyrapone (doses: 200 – 1000 mg and 750 – 4500 mg, respectively) for an average of 4 months (range, 1 – 30), one study showed control (defined as UFC, clinical parameters and morbidity normalization) in 23% of patients with Cushing's syndrome (5/22) in the preoperative period (108). The second study assessed the effect of a triple combination of mitotane, metyrapone and ketoconazole as an alternative to adrenalectomy in 11 patients with severe ACTH-dependent Cushing's syndrome (UFC: 853 – 22605 µg/24 h, reference 10 – 65), 4 patients with CD and 7 with EAS. Significant and rapid (24 – 48 hours) improvements in clinical and laboratory parameters were observed in all cases, with a reduction of UFC from 2737 to 50 µg/24 h (range, 18 – 298) and normalization achieved in 64% of patients. The treatment was initiated with all three drugs simultaneously: 2250 mg/day metyrapone, 800 mg/day ketoconazole, and 3000 mg/day mitotane. The doses were adjusted according to clinical severity, UFC, and tolerability (119).

### Combined use of drugs targeting corticotrope tumors and adrenal steroidogenesis inhibitors

There is few published data concerning combined use of cabergoline with ketoconazole. One of the first studies on this subject was published in 2010 (83), involving 12 patients with CD (microadenomas) and active disease after surgical failure. The protocol consisted of initiating treatment with 1 mg/week of cabergoline (0.5 mg twice/week), with monthly adjustments of 1 mg/week according to UFC or up to 3 mg/week (1.5 mg twice/week) for 6 months. UFC normalization occurred in 3 patients (25%). In the remaining 9 uncontrolled patients, low dose of ketoconazole (100 mg/day) was added, with monthly adjustments of 100 mg or up to 400 mg/day (200 mg twice/day). Control was achieved in 6/9 patients (66%) using both drugs. The remaining 3 patients, those with higher pre-treatment UFC levels (range, 882 – 991 µg/24 h, reference 10–90), presented a lower response to cabergoline only. Thus, after 12 months, UFC was controlled in 75% of patients with cabergoline only or cabergoline + ketoconazole, without escapes during treatment (83). A recent prospective study also assessed this combination therapy in 14 patients with CD by comparing two different regimens: cabergoline followed by ketoconazole (n = 6) vs. ketoconazole followed by cabergoline (n = 8) (84). Cabergoline was initiated at doses of 0.5 – 1 mg/week up to a maximum of 3 mg with the objective of normalizing UFC and late-night salivary cortisol (NSC). Alternatively, ketoconazole was initiated at a dose of 200 mg/day up to a maximum dose of 600 mg/day. Regimens were maintained for 6 months. After this treatment period, patients who achieved UFC and NSC normalization were maintained on monotherapy; patients with values outside of normal ranges received combination therapy (either with cabergoline or ketoconazole) for an additional 6 months. In the first 6 months, none of the patients achieved control (UFC and NSC normalization) with cabergoline, although 33% of patients (2/6) achieved UFC normalization. Ketoconazole monotherapy induced control in 62.5% of patients (5/8) at 6 months. UFC normalization occurred in 79% of patients who were treated with combination therapy, and no differences were observed between the two different combination regimens (84).

Another study evaluated a triple combination regimen with pasireotide, cabergoline and ketoconazole in the preoperative period of 17 patients with CD (99), with the objective of normalizing UFC in 80 days. The treatment was initiated with 100 µg SC pasireotide, 3 times per day, up to a maximum of 250 µg SC, 3 times per day. The increases were performed according to UFC, and the treatment continued for 30 days. After this period, for patients who did not achieve control, cabergoline was added to the treatment at a dose of 1.5 mg/week up to a dose of 4.5 mg/week for 30 days. Following this period, patients who did not exhibit a normalized UFC in 60 days with pasireotide+cabergoline received ketoconazole at a dose of 600 mg/day for 20 days. Overall, control was achieved in 29% of patients with pasireotide monotherapy, 47% of patients with pasireotide+cabergoline, and in 88% of patients with the triple combination. However, the achieved control of 88% does not necessarily mean that all of these patients needed the triple combination. The side effects were worsening of HbA1c levels from 5.8±0.2 to 6.7 ± 0.3% (*p* < 0.01) and reduction of IGF1 to levels lower than reference in 53% (9/17) (99). Although a high rate of control was obtained over a short-term period, this was a small study with an unusual and expensive regimen requiring rapid adjustments, with a potential for significative adverse events. Moreover, the outcome of monotherapy with cabergoline and with ketoconazole was not tested. For this reason, more studies enrolling a larger number of patients and, perhaps, exploring different therapeutic sequences are necessary.

### Other medications and perspectives

Temozolomide is an oral, imidazotetrazine alkylating chemotherapy agent used primarily for the adjuvant treatment of cerebral gliomas. This compound has been increasingly used to treat aggressive/atypical pituitary adenomas and pituitary carcinomas. The antitumor activity of temozolomide occurs through its active form, monomethyl-triazen-imidazole-carboxamide (MTIC), which promotes DNA methylation. This action can be neutralized by the presence of the DNA repair enzyme O-6-methylguanine-DNA methyltransferase (MGMT), an effect that can be assessed by immunohistochemistry or real-time reverse transcription PCR (RT-PCR) on tumor tissue. Although most patients who are responsive to temozolomide show low expression of MGMT, recent studies have shown some cases of dissociation between enzyme expression and drug action (120,121). Temozolomide is usually prescribed in monthly 5-day cycles at a dose of 200 mg/m^2^/day (tablets, 5/20/100/140/180/250 mg). A review study showed clinical improvement (hormonal and tumor reduction) in 50% (4/8) of patients with corticotrope adenomas and in 83% (5/6) of ACTHproducing pituitary carcinomas (122). Temozolomide is generally well tolerated, and the most significant side effects are leukopenia and thrombocytopenia. This medication is reserved for refractory and aggressive cases, not only ACTH-producing tumors, and tumor escape from its salutary effects may occur (121). It is not approved for CD in either Brazil or the USA, and it is rather costly.

Retinoic acid is known to have *in vitro* effects on corticotrope tumors and in an animal model of CD (canine) (123-125). The action of retinoic acid is likely mediated by a decrease in ACTH secretion and proopiomelanocortin (POMC) synthesis, as demonstrated in a murine corticotrope cell line by the inhibition of POMC transcription (123). In addition, retinoic acid has an antiproliferative action. In a recent prospective, proof of concept study, 7 patients with CD were treated with retinoic acid (tretinoin) at a dose of 80 mg/day for 6 – 12 months. A significant UFC reduction (> 50%) in 71% of patients (5/7) with normalization in 43% of cases (3/7) was observed (126) and the drug was well tolerated. Another study with 16 CD patients showed UFC normalization in 25% of cases with isotretinoin use (20-80 mg/day) for 6-12 months (127).

Other drugs have been studied *in vitro* and in animal models, showing action on corticotrope tumors but still lacking results in patients with CD. These drugs include bexarotene (retinoic acid receptor (RXR) agonist) (72), (α1-adrenergic receptor antagonist) (128) and gefitinib (EGF receptor antagonist) (129).

Osilodrostat (LCI699) is a new developed steroidogenesis inhibitor. This drug was initially described as an aldosterone synthase inhibitor with potential for the treatment of hypertension (130). Osilodrostat is a potent inhibitor of 11β-hydroxylase and 18-hydroxylase (98). In a small proof-of-concept study, 10/11 patients with mild to severe CD achieved UFC normalization after 70 days of treatment with 5 – 10 mg twice per day. The main side effects were fatigue, nausea, headache, and an ACTH level increase of more than 2-fold in 5 cases (43). More recent and prolonged study lasting 22 weeks observed normal UFC in 89.5% (17/19) of CD patients (131).

Levoketoconazole is the 2S, 4R enantiomer of ketoconazole, purified from racemic ketoconazole. In *in vitro* studies, levoketoconazole was shown to be a more potent inhibitor than the 2R,4S enantiomer (132). An open-label, phase III, dose-titration study evaluating levoketoconazole in patients with Cushin's syndrome is ongoing.

Table 1 provides a summary of the drugs used for the treatment of CD.

**Table 1:** Drug treatment in Cushing's disease

Drug	Initial dose	Maximum Dose	Control*	Duration	Observations
**Act on the corticotrope tumor**					
Cabergoline	0.5 mg OR 2x/week	3 mg/week (1-7)	25-40%	18 months (3-60)	Escape from treatment in 18-30%; *off label*
Pasireotide	600 mg SC 2x/day	1800 mg/day	29%	12 months	Frequent hyperglycemia
**Steroidogenesis inhibitors**					
Ketoconazole	200 mg OR 2-3x/day	1200 mg/day	52%	22 months (6-72)	Escape from treatment in 33%; mild common increase in ALT/AST; improves hirsutism; hypogonadism in men
Metyrapone	250 mg OR 3-4x/day	4 -6 g/day	26-75%	4 months	Rebound increase of ACTH: hirsutism/acne, HAS/hypokalemia; N/A
Etomidate	IV bolus 0.03 mg/kg; 0.1 mg/kg/h	0.3 mg/kg/h	100%	7 days (5 h-56 days)	Used in severe cases; hospital use (monitoring)
Mitotane	500 mg OR/day	2-3 g/day	72%	7 months	Frequent side effects; difficult handling; high cost
**Cortisol receptor antagonist**					
Mifepristone	300 mg OR/day	1200 mg/day	60% (glycemia AUC)	24 weeks	Approved for the control of DM in Cushing's syndrome; very high cost; N/A
**Combination therapies**					
Ketoconazole + metyrapone	200/750 mg/day	1000/4500 mg/day	23%	4 months	Metyrapone N/A
Mitotane + metyrapone + ketoconazole	3000/2250/800 mg/day	3000/2250/800 mg/day	100%	< 6 months	Critically ill patients; effect in 24-48 h
Cabergoline + ketoconazole	1 mg/week/100 mg/day	3 mg/week/400 mg/day	75-79%	12 months	Short-term studies
Pasireotide + cabergoline + ketoconazole	100 mg SC 3x/day/1.5 mg/week/600 mg/day	250 mg SC 3x/day/4.5 mg/week/600 mg/day	88%	80 days	> 50% IGF1 reduction; frequent hyperglycemia

* Control commonly defined as the normalization of 24-hour urinary cortisol; OR: oral route; SC: subcutaneous; ALT/AST: alanine aminotransferase/aspartate aminotransferase; ACTH: adrenocorticotropic hormone; AUC: area under curve; DM: diabetes mellitus; IGF1: insulin-like growth factor 1; N/A: not available.

### Radiotherapy

While used less frequently in patients with GH and prolactin-producing pituitary tumors, radiotherapy is still an important option for adjuvant or rescue treatment in patients with ACTH-producing tumors (133,134).

Pituitary radiotherapy for CD is classically indicated as a secondary treatment after surgical failure. Radiotherapy is also used in uncontrolled patients receiving drug treatment, particularly those with residual or non-resectable tumors (e.g., cavernous sinus invasion). Radiotherapy is rarely used as a primary treatment in cases where surgical treatment is contraindicated. A study performed nearly 40 years ago showed better efficacy in the pediatric population than in the adult population and for this reason, radiotherapy has been more utilized in children (135), although this approach is not currently accepted. In addition to hormonal control, another objective of radiotherapy is tumor mass control, either via reduction or stabilization/prevention of growth (i.e., the “oncologic” indication). Usually, the efficacy of tumor mass control is higher than hormonal control, ranging from 83% to 100% (29,134,136).

As hormonal control is initiated at least 6 months after radiotherapy (mean, 18 – 24 months), there is a need for medical treatment in this interval (19,29,134). In other words, patients should not be followed without medication waiting for the effects of radiotherapy. In patients with effective concomitant clinical treatment, the effect of radiotherapy may be assessed by biannual withdrawn of drug treatment to measure cortisol (e.g., UFC, NSC and/or low dose dexamethasone suppression test (LDDST)) (19,133).

Most data regarding the control rates and prevalence of complications of radiotherapy are derived from older studies that used conventional methods. More modern stereotactic radiotherapy techniques have been developed (i.e., fractionated or single dose), which are potentially more effective and induce less morbidity.

Radiotherapy is usually performed on a welldefined therapeutic target, particularly in the context of single-dose stereotactic radiotherapy (i.e., radiosurgery). However, it is occasionally performed without a clear target lesion in CD and radiation may encompass the entire pituitary tissue, provided that a diagnosis of central origin has been confirmed (e.g., ACTH+ pituitary adenoma, previous PO remission, or central to peripheral ACTH gradient at BIPSS). In cases in which the target is well-defined, single dose stereotactic radiotherapy may be performed in lesions up to 4 cm in diameter, although it is recommended to allow a minimum distance in relation to optic structures (nerves and chiasm) of at least 3 – 5 mm. In lesions that are close to these structures, fractionated radiotherapy is safer.

Generally, hormonal control is achieved within 2 – 5 years (mean follow-up period, 5 – 10 years) in approximately 50 – 60% of cases (27,29,136), regardless of the technique. More recent studies performed with radiosurgery showed a remission rate of approximately 50% (45,116,134,137) over a similar period (4 – 8 follow-up years). There is no good evidence of a more rapid effect of radiosurgery in comparison to conventional techniques (136). Some studies recommend drug treatment interruption during radiotherapy sections due to the risk of decreased efficacy (138), but this is a controversial issue.

The main concern regarding radiotherapy is related to potential side effects. The most common is hypopituitarism, which occurs in over 50% of patients in the long-term. Other reported effects are optical lesions (neuritis, 1 – 2%), radionecrosis of the brain parenchyma (< 1%), radioinduced secondary tumors (1.5%) (139), neurocognitive disorders (< 1%), and cerebrovascular diseases (< 5%). However, stereotactic techniques, especially radiosurgery that is focused on the lesion and results in less radiation exposure to adjacent tissues, has shown lower complication rates (range, 0 – 1.3%), with the exception of hypopituitarism (72% in 17 years) (140). It is actually not known whether stereotactic radiotherapy is also associated with an increased risk of secondary tumors since this is a relatively novel treatment modality and long-term dara are lacking.

### Adrenalectomy

Bilateral adrenalectomy is considered a 100% effective hypercortisolism treatment (27,141). The main advantage of this method is the immediate normalization of cortisol levels. Currently, the surgical procedure is performed through a laparoscopic approach with reduced rate of PO complications and reduced time of hospitalization (141). Some cases may present recurrence of hypercortisolism due to a vicarious increase of ectopic adrenal tissue or adrenal residues after incomplete surgical resections (142).

Adrenalectomy is generally indicated as the last therapeutic option in refractory cases following unsuccessfully surgical, drug and/or radiation treatment. This treatment may also be primarily indicated in severe cases of CD, for which rapid clinical resolution is required, as well as in patients with EAS (143). Finally, adrenalectomy can be performed in women of reproductive age who wish to become pregnant without hormonal stimuli and for whom repeated pituitary surgery and, particularly, radiotherapy could induce hypogonadotropic hypogonadism. One criticism brought up by some authors is related to the long time for adrenalectomy indication, exposing the patients for prolonged hypercortisolism and its catastrophic effects. This situation can be mitigated with treatment optimization based on rapid changes in the therapeutic approach, particularly with medical treatment.

The disadvantages of bilateral adrenalectomy are permanent adrenal insufficiency with the consequent need of lifelong gluco- and mineralocorticoid replacement, the risk of acute crisis in stress situations, and the development of corticotrope tumor progression – Nelson's Syndrome (NS) (144).

Corticotrope tumor progression may occur following adrenalectomy in 21% of cases over 3 – 5 years (145). Predictive risk factors for occurrence of corticotrope tumor progression are younger age, no previous radiotherapy, the presence of pituitary tumor residues or invasive tumors, higher UFC levels and mainly ACTH increase in the first year after adrenalectomy (145). However, there has been controversy regarding the ACTH cutoff level indicating increased risk. Some authors suggested that an increase of plasma ACTH levels ≥ 600 or ≥ 1000 pg/mL on the first year after adrenalectomy might indicate tumor progression (144,146). Due to inconsistent data, radiotherapy has not been used as prophylaxis, especially with available pituitary MRI imaging and ACTH measurements that are systematically assessed during the follow-up in order to identify corticotrope tumor progression. Interestingly, although the suggested risk of corticotroph tumor progression has been around 50% (140), many of these patients do not develop clinical features of Nelson's syndrome with mass effect or skin hyperpigmentation. Thus, is seems that clinical Nelson's syndrome is less common than corticotroph tumor progression after bilateral adrenalectomy.

Finally, an old study compared remission rate of pituitary surgery with a unilateral adrenalectomy protocol associated with conventional primary pituitary radiotherapy. This report found a similar result of 64% remission of both strategies (142). However, due to the absence of similar data and a lack of improved surgical remission rates, the clinical applicability of this approach is limited.

### Pregnancy

Pregnancy during active CD is very rare and difficult to handle. The reason for the low prevalence is that hypercortisolism interferes with fertility, particularly due to changes in LH/FSH pulsatility, causing menstrual changes/amenorrhea and anovulation. In addition, an increase in androgen production usually occurs due to ACTH stimulation, which inhibits the normal dynamics of gonadotropins. Perhaps, for this reason, most pregnant patients with CD are carriers of adrenal adenomas (40 – 50%) (147-150).

Although rare, the identification and correct treatment of this condition is important due to the increased risk of materno-fetal complications (> 70%) (147,148).

The most common treatment approaches are pituitary surgery, medical therapy, and bilateral adrenalectomy. Expectant approach is also possible when the Cushing's syndrome diagnosis is made at the end of pregnancy, with careful management of associated comorbidities, such as hypertension and diabetes (148). When treatment is indicated, it is usually performed during or after the second trimester due to the time needed to establish the diagnosis. During the gestation period, most pituitary surgeries have a good outcome, and for this reason, pituitary surgery should be the first treatment of choice. Alternatively, during or after the second trimester, drug treatment may be chosen. In this case, metyrapone has been the most reported drug but it is not available in Brazil. The use of has been reported in few cases without the occurrence of congenital malformations (96). However, ketoconazole has been shown to be teratogenic in animal studies and should only be used when metyrapone is not available or induces side effects. There is a single report of cabergoline use in a pregnant patient with CD (151). In more severe and unresponsive cases, as well as in adrenal-related disease, adrenalectomies have been performed in several patients (147,148) and are generally effective.

**Figure 1:**
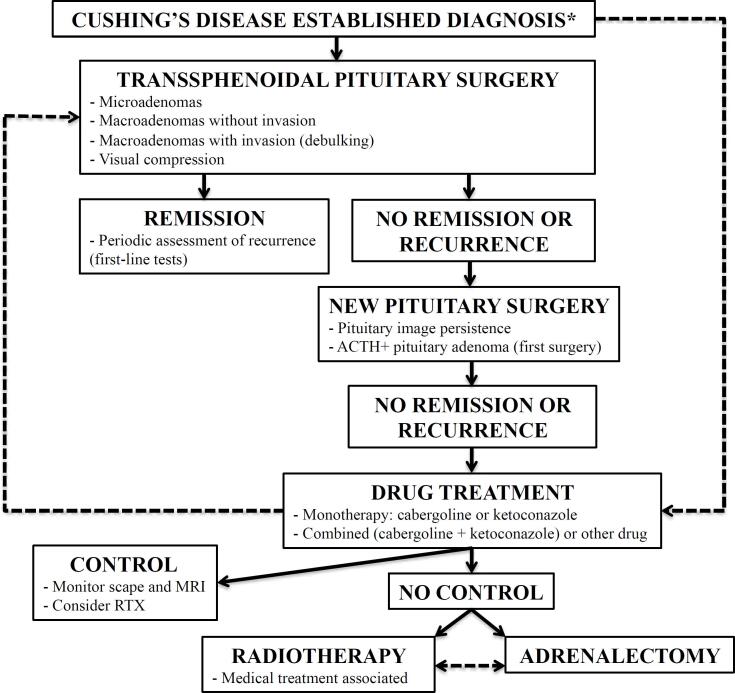
Treatment algorithm for the treatment of Cushing's disease. *Primary clinical treatment may be considered in patients with a contraindication to surgery, those who need to improve preoperative clinical conditions, those who refuse surgical treatment, and in case an experienced surgeon is unavailable. Pituitary surgery should be performed in tertiary centers by experienced surgeons, and patient referral should be considered when these conditions cannot be achieved. Very severe cases may undergo initial bilateral adrenalectomy. RTX: pituitary radiotherapy.

Table 2 summarizes indications for therapeutic options in CD, and Figure 1 shows a proposed treatment algorithm.

**Table 2:** Therapeutic options in Cushing's disease

Type	Control*	Advantage	Disadvantage	Observations
Pituitary surgery	MIC: 70 – 90%	Rapid; direct	Lower remission in invasive macros, and when tumor not visible in image	General treatment of choice
	MAC: 50 – 70%			
Subsequent pituitary surgery	40 – 70%	Possibility of definitive resolution	Lower remission compared to first surgery; increases the risk of cerebrospinal fluid fistula and hypopituitarism	Best suited for patients with persistence of tumoral image
Drug treatment	40 – 100%	Noninvasive; allows patients to undergo surgery if needed	Chronic use; side effects	Mainly indicated in surgical failure, and after RTX
Stereotactic radiotherapy	50 – 60%	Direct treatment	Slow start; side effects (hypopituitarism)	Associated with drug treatment
Bilateral adrenalectomy	100%	Immediate control	Risk of corticotrope tumor progression; permanent gluco- and mineralocorticoid insufficiency	Indicated in severe cases, refractory cases, and when pregnancy is desired

* Control of urine free cortisol; MIC: microadenoma; MAC: macroadenoma; RTX: radiotherapy.
